# Improvement of Endurance of DMD Animal Model Using Natural Polyphenols

**DOI:** 10.1155/2015/680615

**Published:** 2015-03-15

**Authors:** Clementina Sitzia, Andrea Farini, Federica Colleoni, Francesco Fortunato, Paola Razini, Silvia Erratico, Alessandro Tavelli, Francesco Fabrizi, Marzia Belicchi, Mirella Meregalli, Giacomo Comi, Yvan Torrente

**Affiliations:** ^1^Laboratorio Cellule Staminali, Dipartimento di Patofisiologia e dei Trapianti, Universitá degli Studi di Milano, Fondazione IRCCS Cá Granda Ospedale Maggiore Policlinico, Centro Dino Ferrari, Via Francesco Sforza 35, 20122 Milano, Italy; ^2^Sezione di Neuroscienze, Dipartimento di Patofisiologia e dei Trapianti, Universitá degli Studi di Milano, Fondazione IRCCS Cà Granda Ospedale Maggiore Policlinico, Centro Dino Ferrari, Via Francesco Sforza 35, 20122 Milano, Italy; ^3^Ystem S.r.l., Milano, Italy; ^4^U.G.A. Nutraceuticals, Gubbio, Perugia, Italy

## Abstract

Duchenne muscular dystrophy (DMD), the most common form of muscular dystrophy, is characterized by muscular wasting caused by dystrophin deficiency that ultimately ends in force reduction and premature death. In addition to primary genetic defect, several mechanisms contribute to DMD pathogenesis. Recently, antioxidant supplementation was shown to be effective in the treatment of multiple diseases including muscular dystrophy. Different mechanisms were hypothesized such as reduced hydroxyl radicals, nuclear factor-*κ*B deactivation, and NO protection from inactivation. Following these promising evidences, we investigated the effect of the administration of a mix of dietary natural polyphenols (ProAbe) on dystrophic mdx mice in terms of muscular architecture and functionality. We observed a reduction of muscle fibrosis deposition and myofiber necrosis together with an amelioration of vascularization. More importantly, the recovery of the morphological features of dystrophic muscle leads to an improvement of the endurance of treated dystrophic mice. Our data confirmed that ProAbe-based diet may represent a strategy to coadjuvate the treatment of DMD.

## 1. Introduction

Muscular dystrophies (MDs) are a heterogeneous group of disorders characterized by muscular wasting and inflammation that ultimately cause reduction of force and premature death [1]. The role of inflammatory cells is not completely understood but it is known that they could exacerbate muscular wasting, either directly or by secreting mediators, like cytokines and complement's component [2], and by reactive oxygen species (ROS) production [3–5]. ROS were shown to increase membrane permeability in muscle fibres, most likely through lipid peroxidation [6]. Different groups demonstrated that increased ROS production was a primary feature of dystrophic muscle damage and not simply a consequence of muscular degeneration [7, 8]. Others showed that ROS-mediated effect on dystrophic muscles increased protein oxidation, which could cause a wide range of deleterious effects on muscle contractile function [9], both in mdx mice [10] and in DMD patients [11]. It is now known that the intracellular increase in ROS formation typical of the pathological conditions of the MDs is mainly due to mitochondria [12]. Even if the precise mechanism by which oxidative stress causes mitochondria dysfunction is not clear, numerous studies reported that ROS could negatively modify both expression and structural conformation of the proteins that are involved in normal mitochondria functions [13, 14].

In latest years, antioxidant supplementation was shown to be useful in the treatment of different diseases like atherosclerosis, autoimmunitary diseases, diabetes, and chronic diseases including muscular dystrophies [15–18]. The beneficial effects of antioxidants were correlated with their ability to reduce oxidative stress, deactivate nuclear factor-*κ*B (NF-*κ*B) pathway, and vascular effects like vasodilatation and antihypertension through NOS pathway [19]. Many natural antioxidants were tested in animal model of muscular dystrophies and these studies reported amelioration in muscle morphology and function [20].

As an example, green tea is rich in antioxidants such as catechins and also in minerals and vitamins so that it was demonstrated to be useful in the reduction of the risk of cardiovascular diseases and of fibrosis development [21]. Interestingly,* in vivo* studies on mdx mice fed with antioxidants derived from green tea showed reduced signs of muscular damage and ameliorated hydroxyl radicals content, oxidative stress, and fibrosis in treated muscles [22]. According to the emerging role of vitamins in the prevention of chronic diseases [23], Murphy and Kehrer observed similarities between the development of pathological signs in muscular dystrophies and the pathology of muscles exposed to oxidative stress in vitamin E deficiency [24]. Messina and coworkers demonstrated that a synthetic vitamin E analogue, IRFI-042, possessing strong antioxidant properties, improved mdx muscle function and reduced the activation of NF-*κ*B [25]. NF-*κ*B is a key regulator of several genes such as the proinflammatory cytokine TNF-*α* [26] and matrix metalloproteinases [27]. In this sense, Kumar and Boriek showed that passive stretch of mdx diaphragm increased activation of NF-*κ*B, which was attenuated by the antioxidant N-acetylcysteine [28]. At the same time, it was demonstrated that systemic administration of the NF-*κ*B inhibitor curcumin stimulated muscle regeneration after traumatic injury, suggesting a beneficial effect of curcumin supplementation in alleviating dystrophic signs [29]. A possible role for coenzyme Q10 (CoQ10) in the development of different diseases was investigated: especially in cardiovascular pathologies, relatively low levels of CoQ10 were assessed in myocardial tissue [30]. More recently, CoQ10 was added to prednisone therapy in DMD patients, increasing muscle strength [31]. Similarly, carnitine held much promise in neural disorders, allowing osmoprotection and modulating immune and inflammatory responses [32]. Several works showed that this compound regulated lipid metabolism in DMD patients, restoring muscle membrane fluidity [33], decreasing glucose oxidation, and reducing fatty acid [34]. Following previously published data [35], Fogagnolo and colleagues demonstrated that mdx mice fed with docosahexaenoic acid (DHA) decreased plasma creatine kinase levels and myonecrosis, reducing inflammatory area and the levels of TNF-*α* [36]. In the end, another natural flavonoid, the baicalein, was used as a potent anti-inflammatory agent to diminish the concentration of free radicals [37, 38].

Palomero et al. showed that muscular fibres during exercise produce ROS [39]. Interestingly, Reid et al. proposed a correlation between ROS levels and force production. They showed that the maximum force was achieved by unfatigued skeletal muscle when exposed to low levels of oxidants. As either an increase or a reduction in ROS levels determined a reduction in muscle force, they suggested that there was an optimal redox state for force production [40]. Reid proposed that ROS could affect muscle force production by oxidation of contractile and excitation-contraction (E-C) coupling proteins [41] and the role of ROS in mediating muscle fatigue was demonstrated by treatment with antioxidants [42, 43]. Recently Renjini et al. showed that oxidative damage in muscular dystrophy correlates with the severity of the pathology [44] while Selsby and collaborators proved that the overexpression of the antioxidant enzyme catalase improved muscle function in the mdx mouse, especially the resistance to fatigue [45]. Following these promising evidences, several clinical trials started using antioxidants in DMD patients. However, the results were disappointing due to a number of factors, which could account for the negative outcome [7]. First of all, DMD patients were chosen at an advanced stage of the disease, when significant muscle fibre loss had already occurred. Unfortunately, antioxidants would be expected to either reduce or prevent muscle damage and degeneration but not to replace lost fibres. Moreover, the antioxidants used in these trials—such as superoxide dismutase (SOD), vitamin E, and selenium—were not membrane-permeant and were ineffective in scavenging intracellular ROS [20]. Furthermore, several works demonstrated that the combination of different polyphenols might enhance their therapeutic effects, due to a synergic effect of different antioxidants or the contemporary targeting of multiple pathologic pathways [17, 46–48].

According to these evidences, we fed mdx mice with a mix of natural polyphenols (ProAbe), constituted by a liquid phase and a solid phase and we evaluated the amelioration of muscle histology, the oxidation damage, and the possible increase of muscle mass and endurance in dystrophic background. Our data confirmed that the treatment with antioxidants could open a new era in treating muscular diseases.

## 2. Results

### 2.1. Muscular Features of mdx Mice

**Figure 1:**
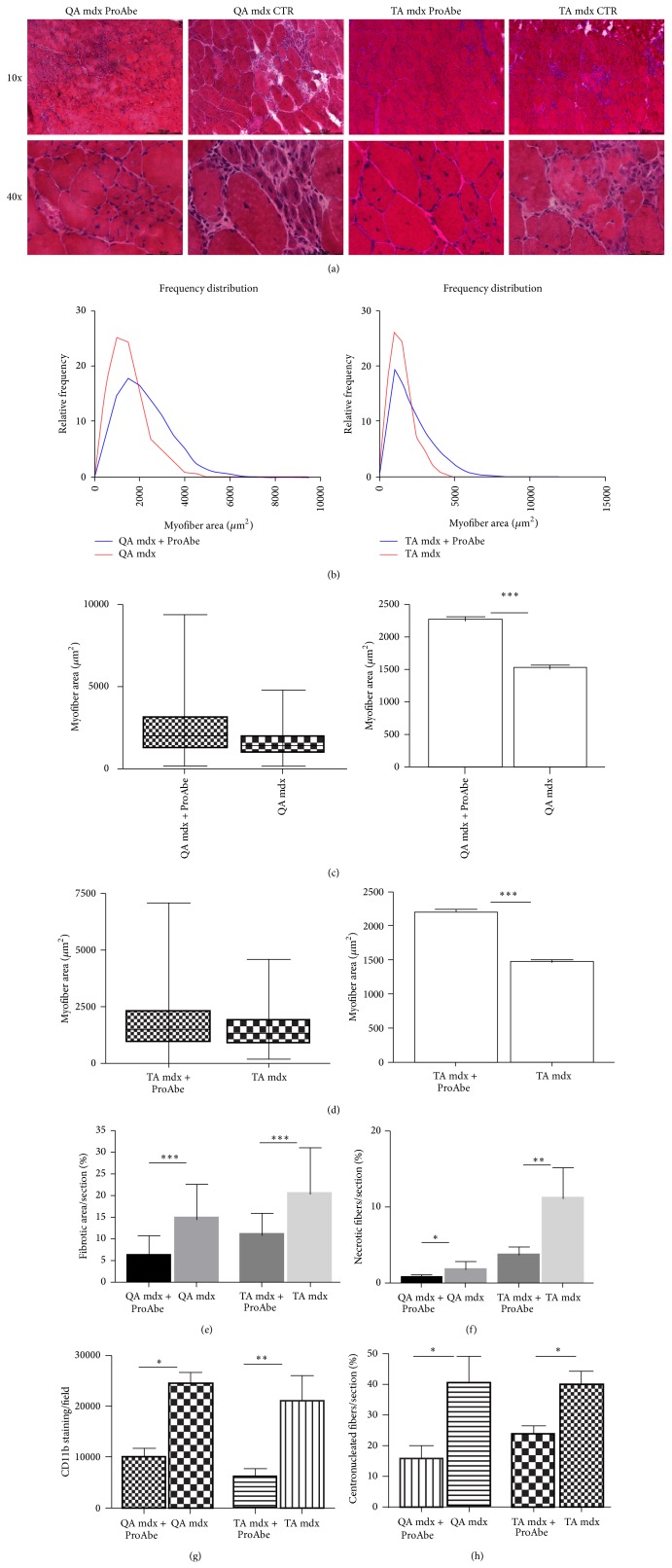
H&E analysis of treated and untreated mice. H&E staining was performed on 10 *μ*m-thick frozen sections from TA and QA muscles (a). In treated mice reduced signs of muscular wasting were observed in comparison to untreated mice. In particular reduced inflammatory infiltrates between myofibers, reduced fat deposition, and reduced necrotic fibers were assessed. In both treated and untreated mice small centronucleated regenerating fibers were observed. For each muscle analyzed with H&E staining we showed the myofiber area and their distribution frequency (b). The curve of TA/QA muscles of treated mice shifted to the right related to untreated mice, thus demonstrating an increase in myofiber area of treated mice (CSA QA: 25° percentile of treated mice 1221,26 and of untreated mice 903,152; CSA TA: 25° percentile of treated mice 1120,39 and of untreated mice 874,068; CSA QA: 75° percentile of treated mice 3107,04 and of untreated mice 1946,06; and CSA TA: 75° percentile of treated mice 2978,59 and of untreated mice 1883,39). Moreover, we indicated the coefficient of variance (graphs showing Min and Max values and mean + SEM) ((c) for QA and (d) for TA). Amelioration of dystrophic phenotype following ProAbe treatment was demonstrated by decrease of fibrosis by measuring area of connective tissue (AM) (e), of the percentage of necrotic fibers (f), of macrophage infiltration area (CD11b staining) (g), and of the number of centronucleated myofibers per section (h).

Fibrosis is considered the most devastating consequence of the progression of disease in DMD patients: due to the lack of dystrophin, satellite cell proliferation cannot compensate constant myofiber breakdown so that inflammatory processes that follow muscular necrosis lead to fibrotic remodelling and finally fatty cell replacement. As in DMD children, the muscle pathology progressed in mdx mice as a function of age. This way, we fed 3-month-old mdx mice (*n* = 5) with ProAbe and we performed H&E analysis of muscle sections to verify whether this diet could delay the onset of the pathology. In tibialis anterior (TA) and quadriceps (QA) of treated mice, we observed the presence of degenerating and small centrally nucleated regenerating muscle fibers, such as in untreated mice; however reduced signs of degeneration (consisting in hypertrophic fibers, fiber splitting, and fat replacement) were seen in treated mice versus untreated ones (*n* = 5) (Figure 1(a)). To verify whether antioxidants supplementation could bear an effect on muscle mass we measured cross-section fiber area (CSA) of both treated and untreated mdx mice. We found that the distribution curves of treated mice shifted to the right in comparison to that one related to mdx control group, thus proving that there was a significant increase in fiber CSA in both muscles examined (TA and QA muscles) (*F* test to compare variance was significant for *P* < 0,0001) (Figures 1(b)–1(d)). In particular there was a reduction in the percentage of smaller fibers (CSA of QA in treated 2274 ± 32,59 and untreated mice 1535 ± 20,08; CSA of TA in treated 1681 ± 23,76 and untreated mice 1486 ± 19,44;* t*-test to compare mean was significant for *P* < 0,0001) (Figures 1(c)-1(d)). To better elucidate that ProAbe-dependent increase of muscle size was not due to fibrotic deposition, other morphological features of the muscles of treated mice were measured. We demonstrated a diminished percentage of fibrosis in both muscles treated with ProAbe (treated QA 6,255 ± 0,632 and untreated QA 14,67 ± 0,66 *P* < 0,0001; treated TA 11,29 ± 0,736 and untreated TA 20,62 ± 1,521 *P* < 0.0001) (Figure 1(e)) and we observed that the percentage of necrotic fibers was significantly smaller in both treated muscles (treated QA 0,8944 ± 0,06 and untreated QA 2,015 ± 0,376, *P* = 0,045; treated TA 3,867 ± 0,4884 and untreated TA 11,35 ± 1,926, *P* = 0,0093) (Figure 1(f)). As clearly described by different works [49–51] inflammatory cells in DMD can interact with resident muscular stem cells and cause the fibroadipogenic degeneration of muscular fibers. According to these evidences, CD11b staining was performed to identify macrophage infiltrates as an indicator of muscle inflammation and we assessed a reduction in inflammatory infiltrates both in TA and in QA muscles of treated mice (untreated TA: 21130 ± 4909 and treated TA: 6187 ± 1460, *P* = 0,0154; untreated QA 24610 ± 2217 and treated QA 10080 ± 1559, *P* = 0,0017) (Figure 1(g)). Moreover, we counted the number of centronucleated myofibers—that are the fibers in regeneration—and we demonstrated that their number was smaller in both treated muscles (untreated TA: 39,79 ± 4,571 and treated TA: 23,64 ± 2,833; *P* = 0,0133; untreated QA 40,42 ± 8,645 and treated QA 18,05 ± 3,981, *P* = 0,0369) (Figure 1(h)).

### 2.2. Force Measurement

**Figure 2:**
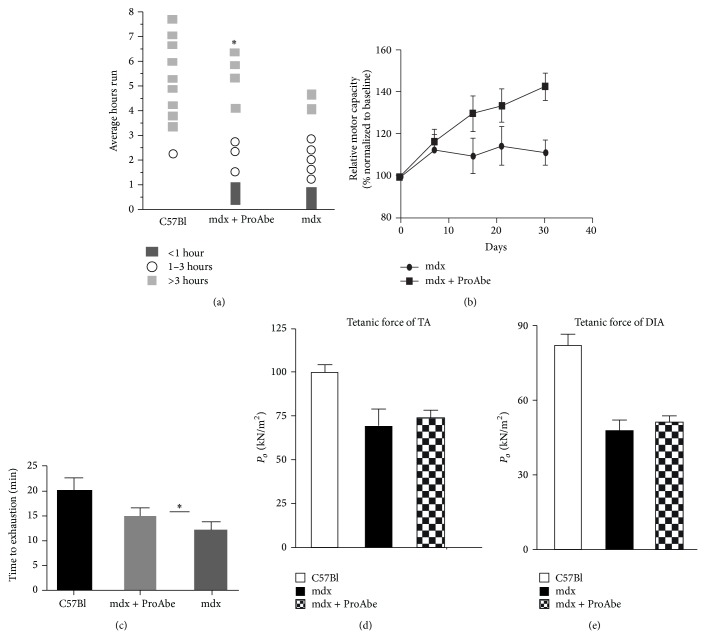
Endurance test. Histogram representing endurance test of treated mice. (a) Each symbol corresponds to a single animal performance; endurance test was repeated once per day for 30 days for each mouse. The average value corresponds to the total amount of time measured during the tests for 30 days. As demonstrated by the average number of hours run mdx-treated mice increased significantly their performance versus untreated mice, even if this value was far from that of C57Bl mice (a). (b) Graph showed relative motor capacity of mice (percentage normalized to baseline) at 4 different time points (each week for 30 days). (c) Graph showed the absolute value of T4 of graph in (b) (that is the average time to exhaustion at T4 (30 days)) that was higher in mdx treated mice related to untreated ones. Tetanic force of TA (d) and DIA (e) was performed in treated and untreated mdx mice.

To verify whether a natural polyphenols diet could improve muscular functionality we first test the endurance of treated and untreated mdx mice (*n* = 10 per each group). In these experiments we found that ProAbe administration significantly increased the endurance ability of mdx mice after 4 weeks of treatment (total average hours run: mdx treated 3,656 ± 0,728 versus mdx untreated 1,844 ± 0,426; *P* = 0,0474) (Figure 2(a)). We measured the total motor capacity relative to baseline performance and we observed an increase of 30% at day 30 of treatment (Figure 2(b)). Dystrophic animals assessed at 30 days of treatment showed an increased tolerance to exercise as demonstrated by the time to exhaustion (mdx treated 14,33 ± 0,817 versus mdx untreated 11,02 ± 0,849; *P* = 0,0117) (Figure 2(c)). Furthermore, we calculated the tetanic force of TA and DIA of treated and untreated mdx (*n* = 5 per each group) and we found a minimum but not significant improvement of the values in treated mdx muscles (Figures 2(d) and 2(e)).

### 2.3. Oxidative State Evaluation

**Figure 3:**
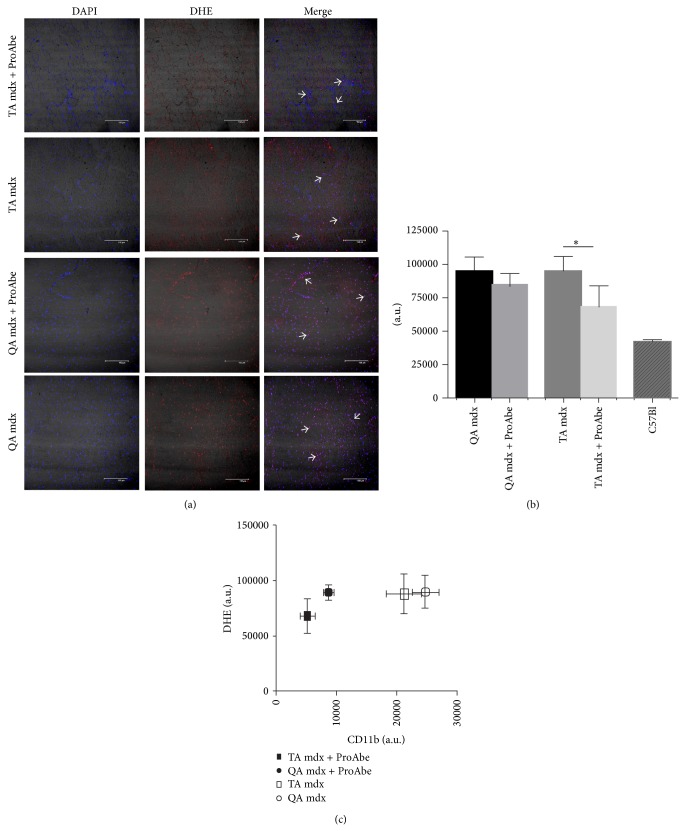
DHE staining and quantification. (a) DHE staining on muscle sections from TA and QA of mdx mice. DHE staining revealed anion superoxide production at nuclear level. Arrows indicated DHE staining on both cellular infiltrate and fibers myonuclei. (b) We quantified DHE staining intensity on muscle section. Histogram showed a reduced production of oxidative damage as measured by DHE staining in TA muscle of mdx-treated mice versus untreated ones (*P* < 0,05). (c) Graph showed the correlation between DHE staining intensity and CD11b+ infiltrate (per field). The mean and SD for each category are plotted. ProAbe treatment reduced CD11b+ infiltrates more than DHE intensity (as shown by right position of untreated mice versus left position of treated mice).

To evaluate whether natural polyphenols diet could influence ROS production we quantified dihydroethidium (DHE) staining in muscle sections from treated and untreated dystrophic mdx mice (*n* = 5 per each group). DHE oxidation by intracellular ROS causes the formation of ethidium bromide, which fluoresces red, once intercalated within DNA. Thus nuclear fluorescence intensity directly correlates with ROS production. We measured DHE fluorescence intensity on sections of TA and QA (Figure 3(a)) and we found that the ProAbe-enriched diet significantly diminished ROS production (TA of treated 68450 ± 7782 and untreated mdx mice 95470 ± 5185; *P* = 0,0277) (Figure 3(b)). In QA muscle of treated mice we observed a similar decrease in ROS production that did not reach statistical significance (QA of treated 82940 ± 3434 and untreated mdx mice 95330 ± 5219; *P* = 0,1183). C57Bl mice were used as control (42380 ± 1378). As shown in Figure 3(a), DHE stained both myofiber nuclei and cell infiltrates: after ProAbe treatment, DHE staining was reduced both in inflammatory infiltrates and in myofiber nuclei (Figure 3(a)). To determine whether the reduction in DHE intensity was related to reduced inflammatory infiltrates (as previously shown in Figure 1(g)), we correlated both data and we showed that the reduction of inflammatory infiltrates was major than the corresponding reduction of DHE intensity (Figure 3(c)). As the number of myofiber nuclei was equivalent, ProAbe treatment influenced more the ROS produced by myofibers than those produced by infiltrating cells in particular macrophages.

### 2.4. Mitochondria

**Figure 4:**
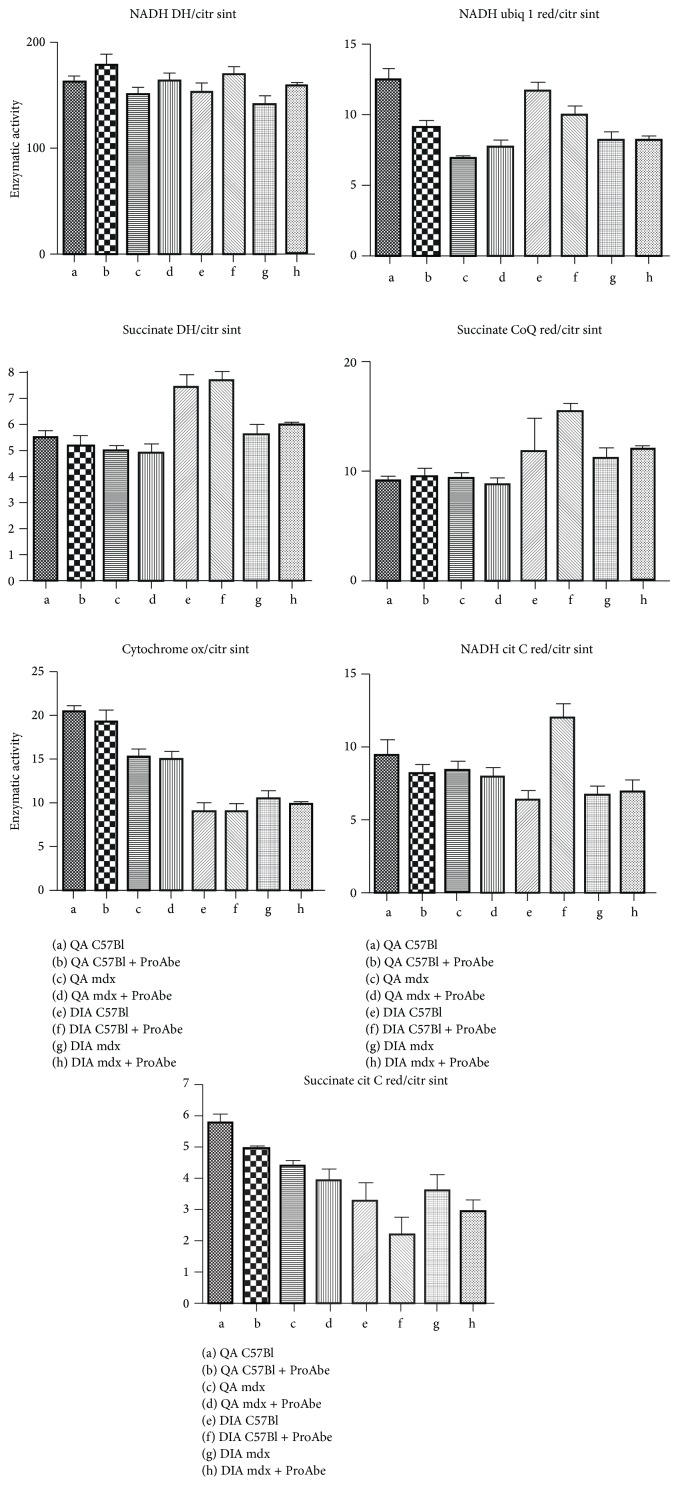
Enzymatic activity of mitochondrial enzymes. Histogram representing enzymatic activity of a wide range of mitochondrial enzymes involved in respiratory chain complexes. No significant differences were evidenced between treated or untreated mdx mice in DIA and QA. The following abbreviations were used in the picture (NADH DH/citr sint: NADH dehydrogenase/citrate synthase; NADH ubiq 1 red/citr sint: NADH ubiquinone 1 reductase/citrate synthase; succinate DH/citr sint: succinate dehydrogenase/citrate synthase; succinate CoQ red/citr sint: succinate CoQ reductase/citrate synthase; Citrate ox/citr sint: citrate oxidase/citrate synthase; NADH cit C red/citr sint: NADH citrate C reductase/citrate synthase; and succinate cit C red/citr sint: succinate citrate C reductase/citrate synthase).

As cited before, we determined whether the effects of ProAbe on ROS production were exerted on mitochondria and we evaluated the activity of mitochondrial enzymes in QA and diaphragm (DIA) of treated and untreated C57Bl (*n* = 5) and mdx mice (*n* = 5) (Figure 4). We demonstrated that there were no significant modifications within the activity of these enzymes in treated mice.

### 2.5. Vascular Features

**Figure 5:**
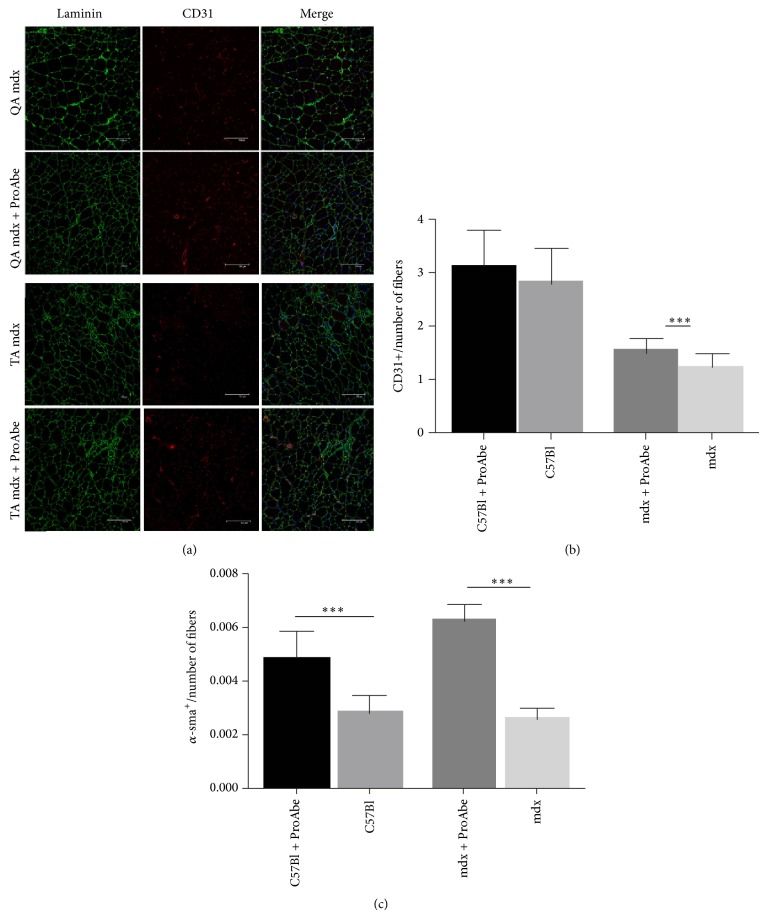
Vascular features of treated mice. (a) Panel representing staining for laminin and CD31 in QA and TA of mdx-treated and untreated mice. (b) The ratio CD31+ vessels/fiber was significant only for TA of mdx mice while (c) the number of a-SMA+ vessels/section was significant for TA of both mdx and C57Bl mice. Analysis of statistical significance was determined by unpaired* t*-test.

To test the influence of antioxidants upon muscle vascular architecture, we performed CD31+, *α*-sma, and laminin immunofluorescence staining on muscle sections from TA and QA of treated and untreated mice (*n* = 5) (Figure 5(a)). We counted the number of CD31+ vessels per fiber (capillaries) and of *α*-sma+ vessels per fiber (arterioles). The increase of CD31+ vessels in treated muscles was statistically significant in dystrophic mice (TA mdx treated versus TA mdx untreated: 1,553 ± 0,059 versus 1,272 ± 0,045; *P* < 0,001), but it was not significant in normal mice (TA C57Bl treated versus TA C57Bl untreated: 3,146 ± 0,1076 versus 2,835 ± 0,1289; *P* = 0,0624) (Figure 5(b)). Therefore, we found that all treated mice had a significant increase in *α*-sma+ vessels per fiber (TA mdx treated versus TA mdx untreated: 7,85 ± 0,2975 versus 3,233 ± 0,2108; *P* < 0,0001; TA C57Bl treated versus TA C57Bl untreated: 6,04 ± 0,34 versus 3,54 ± 0,2; *P* < 0,0001), thus demonstrating an amelioration of muscle perfusion (Figure 5(c)). QA muscles had similar but less significant amelioration of vascular compartment (data not shown).

### 2.6. Stem Cell Mobilization Mediated by ProAbe Treatment

**Figure 6:**
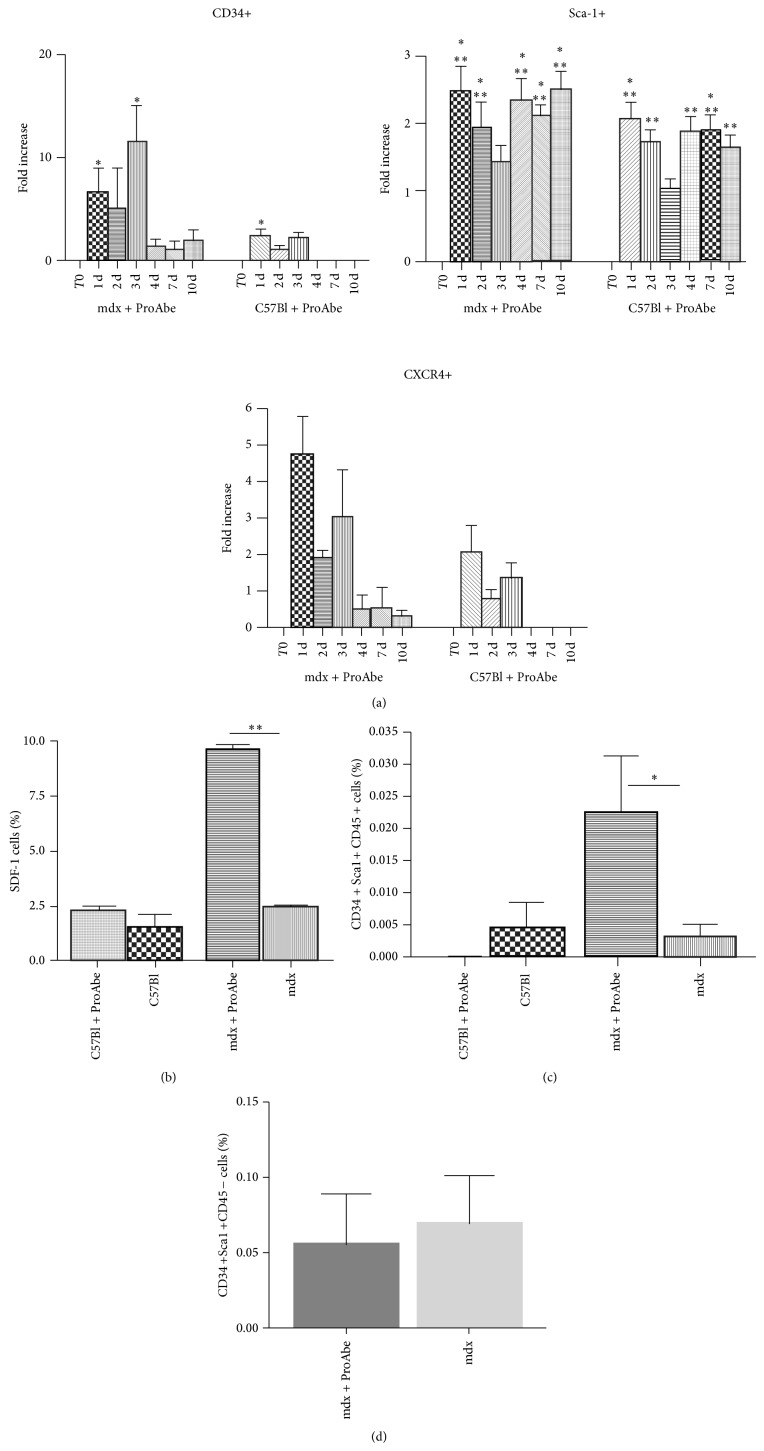
Stem cell mobilization driven by ProAbe. (a) Histograms representing FACS analysis of percentage of stem cell and adhesion marker in PBMCs from treated C57Bl and mdx mice. T0 (before treatment) was used as baseline; bars represented the fold increase of cell's percentage relative to T0. At T24 h, both mdx and C57Bl mice showed an increase in the percentage of peripheral CD34+, Sca-1+, and CXCR4+ cells. After 72 h of treatment the percentage of peripheral CD34+ and CXCR4+ started to decline. After 10 days of treatment the percentage of markers analyzed was similar to T0 levels. (b) FACS analysis, showing the percentage of SDF-1+ cells in QA, was higher in both the mice fed with ProAbe; in particular, in dystrophic mice it was more than 3-fold higher. (c) FACS analysis showing that the percentage of CD34+Sca-1+CD45+ cells isolated from QA was significantly higher only in the mdx-treated mice. (d) FACS analysis showing the percentage of CD34+Sca-1+CD45− cells isolated from treated and untreated mdx mice.

To test the hypothesis that ProAbe could favour the mobilisation of bone marrow stem cells as demonstrated for other natural components [52–54], we collected the blood from C57Bl and mdx mice fed with ProAbe at different time points (*n* = 5 for each strain) and we investigated the expression of different cellular markers by FACS analysis. T0 (before treatment) was used as baseline. We chose CD34, a cell-cell adhesion factor, able to mediate the attachment of stem cells to bone marrow extracellular matrix; CXCR4 that regulates the mobilization of haematopoietic stem cells into the bloodstream as peripheral blood stem cells; and Sca-1 that is a classical stem cell antigen. Following the treatment with ProAbe, we observed a mobilization of stem cells from bone barrow to the peripheral blood (7-fold increase in peripheral CD34+ stem cells at 24 h of treatment related to baseline) and we assessed that the mobilization in the dystrophic mice was higher related to C57Bl mice. Values were normalized on the total number of CD45+ stem cells and the significance of these values was related to the T0 (before treatment) value (% of CD34+ in mdx: T0: 0,8775 ± 0,2893; T24: 4,684 ± 0,6313 with *P* = 0,028; T72 h: 8,717 ± 2,309 with *P* = 0,0104. % of CD34+ in C57Bl: T0: 0,7462 ± 0,2366; T24: 3,554 ± 1,001 with *P* = 0,0245), (% of Sca-1+ in mdx: T0: 27,11 ± 2,053; T24: 64,8 ± 5,408 with *P* < 0,0001; T48 h: 64,95 ± 1,073 with *P* < 0,0001; T4 d: 65,96 ± 3,992 with *P* < 0,0001; T7 d: 64,16 ± 1,016 with *P* < 0,0001; T10 d: 62,82 ± 3,043 with *P* < 0,0001. % of Sca-1+ in C57Bl: T0: 36,95 ± 4,967; T24: 72,25 ± 2,785 with *P* = 0,0003; T48 h: 62,25 ± 0,8039 with *P* = 0,001; T4 d: 67,49 ± 3,772 with *P* = 0,0012; T7 d: 67,3 ± 1,214 with *P* = 0,0003; T10 d: 60,55 ± 1,746 with *P* = 0,0021), and (% of CXCR4+ in mdx: T0: 0,4009 ± 0,1396; T24: 2,805 ± 1,091 with *P* = 0,0425) (Figure 6(a)). Furthermore, we analysed the expression of stromal cell-derived factor 1 (SDF-1), the ligand of the CXCR4, by the cells isolated from QA of treated and untreated mice and we demonstrated that, after 7 days of ProAbe treatment, in mdx mice there was a significant mobilization of SDF-1+ stem cells from bone marrow (QA of treated mdx 9,65 ± 0,25 versus QA of untreated mdx 2,5 ± 0,1; *P* = 0,0014; QA of treated C57Bl 2,25 ± 0,25 versus QA of untreated C57Bl 1,5 ± 0,5) (Figure 6(b)). In addition skeletal muscle from mdx treated mice contained a higher fraction of a cell population CD34+Sca-1+CD45+ characterized by bone marrow origin and stemness (QA of treated mdx 0,026 ± 0,008645 versus QA of untreated mdx 0,00323 ± 0,001862; *P* = 0,0419) while in C57Bl the two fractions were similar (Figure 6(c)). In fact a subpopulation of cell isolated from skeletal muscle shared few peculiarities of bone-marrow-derived HSCs (expressing Sca-1 and c-kit); however muscle HSCs do not express CD45 while HSC derived from bone marrow express this antigen [55]. This way, the population CD34+Sca-1+CD45− representing resident stem cell population was analyzed in QA of treated and untreated mice and we found that it was unchanged (Figure 6(d)).

## 3. Discussion

Unfortunately extensive researches were not enough to build up efficacious strategies not only to correct the primary defect of DMD but also to alleviate the downstream pathologies. In the latest years, it was clear that membrane fragility caused by the absence of dystrophin led to inflammation and fibrosis of muscles so that several antioxidants and nutritional supplements were studied to inhibit the activity of certain molecules (NF-*κ*B, TNF-*α*, and TGF-*β*) and, subsequently, to modulate these devastating phenomena. Several works demonstrated that, following supplementation in mdx mice, natural compounds were able to reduce fibrosis [22] and to ameliorate muscle strength [31]. Furthermore, they exerted antioxidant activity [25] and regulated the inflammatory cells [32] and lipid metabolism [33]. It is known that ROS could exacerbate muscular damage by oxidizing membrane phospholipids and proteins, thus increasing membrane permeability and leakage [56, 57]. As expected, after ProAbe treatment, we observed a reduction in necrotic fiber and in mononuclear cells' infiltration among intact fibers, together with reduced fibrosis deposition. Furthermore small centronucleated fibers were diminished according to a reduction of percentage of smallest fibers and, conversely, a general increase in muscular mass (as shown by right-shift of frequency distribution curve) demonstrating that an antioxidant-based diet could modulate muscular damage. We also demonstrated that in treated mice there is a reduction of muscular anion superoxide production and consequently of DNA oxidation as observed by quantification of DHE staining, confirming that ProAbe supplementation could influence the oxidative state of the muscle. In addition, ProAbe could modulate ROS production both by reducing inflammatory infiltrate or by regulating inflammatory cells activity [58]. Skeletal muscle mass is regulated by a balance between protein synthesis and degradation, which is sensitive to many environmental triggers such as mechanical load, growth factors (myostatin), hormones (glucocorticoid), inflammatory cytokines (TNF-*α* and IL-6), oxidative stress (ROS and NO), metabolic stress (ATP levels), and nutrient availability. Even if we did not demonstrate an improvement of tetanic force in examined muscle, we found an amelioration in endurance test in mice fed with an antioxidant-rich diet. According to these data, it was previously demonstrated that resistance to fatigue was increased by a diet rich in antioxidants, probably because of the reduction of calcium overload [45, 59]. Successively, we tried to correlate the amelioration of endurance and the diminished production of ROS with possible role of mitochondrial enzymes, but we did not assess any significant difference in the activity of these enzymes between treated and untreated mice. On the other hand, as muscular performance could depend on vascular supply to each fibre, we demonstrated an increase in arteriolar supply by counting *α*-sma+ vessels and capillaries per fibers in all treated mice. The mechanism by which antioxidants are involved in vascular remodelling is not completely clarified, but it is known that polyphenols, like resveratrol, could protect NO from inactivation favouring its biologic activities [19].

Since the work of Gussoni that firstly described the presence of haematopoietic stem cells (HSCs) in adult skeletal muscle [60], it was demonstrated that these cells could be recruited according to specific molecular cues from bone marrow to muscle, where they actively participated in muscle regeneration [61]. Several papers showed that transplantation of bone-marrow-derived HSCs in animal models of DMD contributed not only to amelioration of skeletal muscle [62, 63] but also to endothelial cell formation [55]—probably due to their residing perivascular niche [64]. According to these evidences, transplantation of bone-marrow-derived HSCs obtained preliminary but promising results in DMD patients [65, 66]. Our data indicated an increase of the CD34+Sca-1+CD45+ stem cells whereas the resident population of CD34+Sca-1+CD45− remained unchanged suggesting a specific mobilization of bone-marrow-derived stem cells. These cells could be involved in the amelioration of the morphological features that we found in treated animals. Currently, mobilized cells are also the preferable and major source of stem and progenitor cells harvested for autologous and allogenic transplantations because of the higher yield of these cells, leading to faster engraftment and decreased procedural risks compared with harvested BM cells. Even if stem cell mobilization spontaneously takes place after tissue damage [67, 68], several enhancers were used for clinical purposes to obtain faster and significant results. It is known that granulocyte-colony stimulating factor (G-CSF) triggers stem cell mobilization [69] while SDF-1 attracts stem cells allowing their extravasation [70]. The effects of these molecules were studied in DMD blood [71, 72], but, unfortunately, they were associated with severe adverse effects so that they were not feasible for human prolonged treatment [73]. However, more recently different works described the emerging role of natural compounds in mediating such kind of events [52–54]. Interestingly, as we observed a mobilisation of HSCs from bone marrow to peripheral blood in treated animals, we suggested that ProAbe could favour the migration of bone-marrow-derived progenitors and their participation in muscular regeneration and endothelial formation. Similar to the data reported by Brzoska and colleagues [74], we showed that the augmentation of SDF-1+ stem cells was associated with lower fibrosis rate and an improvement of muscle regeneration. According to our results and recently published data [75–77], we speculated that mobilized stem cells could participate in the formation of new myofibers while, alternatively, those cells could participate in angiogenesis in treated animals.

In conclusion an antioxidant-rich diet seems a promising approach to coadjuvate therapies concerning untreatable disease like muscular dystrophy, thanks to their ability to improve histological and functional features of muscles and to enhance the recruitment of stem cells. As it was shown that combination of different polyphenols/antioxidants could enhance their effects [17, 46–48], ProAbe could be useful for the administration of a lower dose of each antioxidant, thus limiting all the possible adverse events [78]. In addition, although the precise mechanisms of action of the majority of these compounds is not well understood, we suggested that ProAbe could be a feasible approach in addition to standard therapy as it can exert functional and morphological ameliorations in dystrophic muscles (increased CSA of myofibers, decreased amount of inflammatory cells and necrotic fibers, and restoration of muscle force) to counteract degeneration, thus allowing a reduction of necessary dose of chronic drugs and limiting their known side effects.

## 4. Material and Methods

### 4.1. Animal Ethics Statement

Procedures involving living animals were conformed to Italian Country law (D.L.vo 116/92 and subsequent additions) and approved by local ethics committees. This work was authorized by the National Institute of Health and Local Committee, Protocol number 6/13-2012/2013. Three-month-old normal (C57Bl) and dystrophic (mdx) mice were provided by Charles River (Calco, Lecco, Italy). The weight of C57Bl was approximately 19 g while the weight of mdx was 25 g. Animals were caged with comfort and safety, in controlled ambient (12-hour light, 12-hour dark) at a temperature between 21°C and 24°C: they were able to move freely within them and had access to clean water and food. After one month of treatment, mice were sacrificed by cervical dislocation according to Italian country law.

### 4.2. ProAbe Composition

The liquid formulation provides DHA plus derived from purified fish oil, deodorized and scented with lemon, and natural vitamin E. The powder formulation provides curcumin conveyed in phytosomes, coenzyme Q10, acetyl-L-carnitine, green tea extract, extract of* Scutellaria*, and vitamin C, whose functions in modulating dystrophic phenotype have been described above [21–23, 28, 31, 33, 34, 36, 38]. ProAbe was provided by Ystem (Milan, Italy) in collaboration with U.G.A. Nutraceuticals (Gubbio, Perugia, Italy).

### 4.3. Dosage

The recommended daily dosage for human is 1000 mg of curcumin; 750 mg of acetyl-L-carnitine; 200 mg of coenzyme Q10; 100 mg of green tea extract; 105,3 mg of extract of* Scutellaria*; 1250 mg of DHA; and 36 mg of vitamin E. Mice were proportionally fed (daily) with the appropriate quantity of ProAbe (human 70 kg; mouse 20 g) for one month for histological and endurance experiments while for mobilization experiments different time points were considered as described in detail in the next section.

### 4.4. FACS Analysis of Blood-Derived Murine Cells

Peripheral blood (100 *μ*L) from the retroorbital sinus was taken from each mouse and the samples were lysed to allow cytofluorimetric studies. Blood samples were collected at different time points: T0 (before ProAbe administration) and T24 h, T48 h, T72 h, T4 days, T7 days, T10 days, and T14 days following ProAbe administration. For five-colour flow cytometry cells were incubated with 10 *μ*L primary antibodies against CD34 FITC, SCA1 PE (BD Biosciences, San Diego, CA, USA), CD184 (CXCR4) APC (Miltenyi Biotech, Bologna, Italy), and CD45PE-Cy7 (eBioscience, San Diego, CA). QA were weighed and washed several times in PBS, finely minced with scissors, and incubated at 37°C for 45 minutes with 1 mg/mL collagenase type IA (Sigma-Aldrich), 80 *μ*g/mL DNase I (Roche), and Trypsin 2,5% (1 : 3) (Gibco) in Dulbecco's modified Eagle's medium (Invitrogen). Most of the skeletal muscle stem cells were released from the tissue after this step. The cell extract was filtered with a 70 *μ*m nylon mesh (BD Biosciences, Immunocytometry Systems, Mountain View, CA) and labelled for FACS analysis. After each incubation, performed at 4°C for 20 min, cells were washed in PBS 1% heat-inactivated FCS and 0.1% sodium azide. Isotype-matched immunoglobulins were added to each control sample. The cells were analysed using the Cytomics FC500 and CXP 2.1 software (BC, Beckman-Coulter). Each analysis included at least 50000–200000 events for each gate. A light-scatter gate was set up to eliminate cell debris from the analysis. The percentage of positive cells was assessed after correction for the percentage reactive to an isotype control conjugated to relative fluochromes. Bone marrow was collected from treated and untreated mdx and C57Bl mice by flushing femurs and tibias with saline solution and red blood cells were lysed with ammonium chloride. Nucleated cells were labelled in phosphate-buffered saline (PBS), 2% fetal calf serum (FCS) for 45 min at 4°C with CD184 (CXCR4) APC and SDF-1 antibody (R&D Systems). Goat anti-mouse 647 was used as secondary antibody.

### 4.5. Immunohistochemistry

From both treated and untreated mdx mice DIA, QA and TA muscles were removed, frozen in liquid nitrogen-cooled isopentane, and sectioned on cryostat. We performed histological analysis on QA and TA as these muscles show similar level of dystrophic degeneration in mdx mice. Furthermore we investigated DIA muscle as it is the muscle that mostly recapitulates human pathology. Serial sections of 10 *μ*m in thickness were stained with H&E and AM. Images were captured with LEICA AS LMD optical microscope. For immunofluorescence analysis, sections were incubated with antibodies: rabbit anti-laminin (1 : 50; Abcam), rat anti-CD31 (1 : 100; BD), anti-*α* smooth muscle actin (1 : 50 Sigma), and anti-CD11b (1 : 50, R&D). The slides were analysed using a fluorescent microscope (LEICA DMIRE2) and a confocal microscope (LEICA TCS-SP2). For quantitative analysis ImageJ Software (NIH) was used. For CD11b and DHE correlation study serial sections were utilised.

### 4.6. Endurance Test and Tetanic Force

Endurance performance test was conducted as previously described [79]. Briefly, 10 mice were placed on the belt of motorized treadmill (Columbus Instruments). The treadmill was run at an inclination of 0° at 5 m/min for 5 min, after which the speed was increased 1 m/min every minute. The test was then stopped when the mouse remained on the shocker plate for 20 s without any attempt to reengage the treadmill, and the time to exhaustion (expressed in minutes) was determined. This test was performed daily for 30 days [80, 81]. Regarding tetanic force measurement, the experiments were performed as previously described [79]. Relative motor capacity was analyzed at 4 different time points (each week for 30 days) while time to exhaustion at T4 (30 days).

### 4.7. Dihydroethidium Staining for ROS Detection

Dihydroethidium (DHE) is a commonly used indicator of ROS production, both* in vitro* [82] and* in vivo* [83]. DHE analysis was performed as previously described [84]. Briefly, ROS production was measured by incubating TA and QA cross-sections with 5 *μ*m DHE in PBS at 37°C for 30 min. DHE intensity was analyzed by confocal microscope and quantified by counting the number of pixels exceeding a specified threshold, which was set in order to eliminate interference from any background fluorescence using ImageJ software.

### 4.8. Mitochondria

We collected DIA and QA of mdx and C57Bl mice fed with ProAbe and we prepared these samples for further analysis as earlier described [85]. Mitochondrial respiratory chain enzyme and citrate synthase activities were measured spectrophotometrically by described assays [85]. The specific activity of each complex was normalized to that of citrate synthase.

### 4.9. Statistical Analysis

Data were expressed as means ± SD. The fibres' counting in mdx mice was compared by Student's* t*-test. To compare multiple group means, one-way analysis of variance (ANOVA) was used. When only two groups were compared, the* t*-test was applied assuming equal variances. The difference among groups was considered significant at *P* = 0,05.
